# Physical events occurring during the cryopreservation of immortalized human T cells

**DOI:** 10.1371/journal.pone.0217304

**Published:** 2019-05-23

**Authors:** Julie Meneghel, Peter Kilbride, John G. Morris, Fernanda Fonseca

**Affiliations:** 1 Asymptote, General Electric Healthcare, Histon, Cambridge, United Kingdom; 2 UMR GMPA, AgroParisTech, INRA, Université Paris Saclay, Thiverval-Grignon, France; Martin-Luther-Universitat Halle-Wittenberg, GERMANY

## Abstract

Cryopreservation is key for delivery of cellular therapies, however the key physical and biological events during cryopreservation are poorly understood. This study explored the entire cooling range, from membrane phase transitions above 0°C to the extracellular glass transition at -123°C, including an endothermic event occurring at -47°C that we attributed to the glass transition of the intracellular compartment. An immortalised, human suspension cell line (Jurkat) was studied, using the cryoprotectant dimethyl sulfoxide. Fourier transform infrared spectroscopy was used to determine membrane phase transitions and differential scanning calorimetry to analyse glass transition events. Jurkat cells were exposed to controlled cooling followed by rapid, uncontrolled cooling to examine biological implications of the events, with post-thaw viable cell number and functionality assessed up to 72 h post-thaw. The intracellular glass transition observed at -47°C corresponded to a sharp discontinuity in biological recovery following rapid cooling. No other physical events were seen which could be related to post-thaw viability or performance significantly. Controlled cooling to at least -47°C during the cryopreservation of Jurkat cells, in the presence of dimethyl sulfoxide, will ensure an optimal post-thaw viability. Below -47°C, rapid cooling can be used. This provides an enhanced physical and biological understanding of the key events during cryopreservation and should accelerate the development of optimised cryobiological cooling protocols.

## Introduction

Cryopreservation is a key enabling technology contributing to the delivery of cell therapies to the clinic. However, many details of critical, cellular responses to cryopreservation stresses are not well understood, which limits the pace of development of improved and efficient cell preservation protocols. A significant area concerns the formation of intracellular ice which is, typically, a lethal event for the cell [1]. During equilibrium cryopreservation of a cell suspension, where slow cooling in the presence of a cryoprotectant such as dimethyl sulfoxide (DMSO) is used, ice forms first in the extracellular compartment. This effectively removes water and produces a two-phase system of ice and a residual, freeze-concentrated solution of suspending medium including cryoprotectant and cells [2, 3]. The osmolality of this freeze-concentrated solution increases as the temperature is reduced and more ice forms. As slow cooling progresses the suspended cells will shrink as they lose water to try to remain in osmotic equilibrium with the extracellular solution. Thus, the cells are able to avoid intracellular ice formation. If the cooling rate is increased, a temperature will be reached where cellular water loss is not rapid enough to effectively reduce the increasing osmotic gradient between cells and suspending solution (non-equilibrium freezing). At this point the remaining water within the cell can form lethal intracellular ice [4]. Understanding more about the physical state of the intracellular compartment of cells that avoid intracellular ice formation during equilibrium cryopreservation is clearly of value for optimising the technology and the freezing protocols.

Vitrification, or glass transition, occurs when a liquid begins to behave as a solid during cooling, with minimal change in thermodynamic state variables such as pressure, volume, internal energy, and entropy [5]. Below the glass transition temperature viscosity exceeds 10^12^ Pa.s [5]. Vitrification does not involve an abrupt entropy change or an exotherm, as seen with freezing, but there are changes in thermodynamic response variables such as heat capacity and thermal expansivity. Consequently, a glass transition can be detected by differential scanning calorimetry (DSC), recording a change in heat capacity [6].

Intracellular vitrification is, however, more complex than in simple, bulk liquids as the interior of the cell contains a crowded, colloidal suspension. In previous studies of equilibrium cooling of microorganisms in their natural environment, and under conditions relevant for cryopreservation, we have shown that the intracellular compartment becomes increasingly viscous and eventually undergoes a colloidal-like glass transition with continued cooling [6, 7]. Similarly, mammalian cells in suspension that are exposed to an osmotic stress, dehydrate and shrink and the packing density of proteins and organelles in the intracellular compartment increases before undergoing a glass transition, analogous to a colloidal glass transition [8]. A colloidal glass has the properties of a dense suspension of colloidal particles rather than that of a molecular glass and behaves like a molecular sieve [9], allowing the free passage of small molecules whilst restricting the diffusion of larger ones.

These widespread vitrification events will be concurrent with the impact of lowered temperature on individual cell components, such as cell membrane lipid phase transition and reduced metabolic activity. However, the temperature of this colloidal-like glass transition has not previously been reported for mammalian cells, and its influence, together with that of related, biological events, on cryopreservation outcomes is not known. Typical cryopreservation protocols for mammalian cells cool at a controlled rate to some arbitrary endpoint, such as -80°C or below the extracellular glass transition. Optimising this endpoint temperature will allow to develop more efficient, consistent, and optimal cell cryopreservation protocols.

In this study we have used Jurkat cells, an immortalized line of human T lymphocyte cells, as an investigative system of relevance to cryopreservation and regenerative medicine. The physical events occurring in Jurkat cells samples during conventional cryopreservation were measured. A simple binary system comprising a cryoprotectant solution and protein at different concentrations was also used to model elements of an intracellular compartment during cryopreservation, to better understand the basic physics of the physical events observed in cell samples. This data was put in perspective to viable cell counts and cellular, metabolic activity, to link physical events and biological outcomes for optimising equilibrium-freezing protocols.

## Materials and methods

### Cells and cell culture

Jurkat cells were acquired from Sigma-Aldrich, Gillingham, UK (#88042803, Clone E6-1, TIB-152) [10, 11] and cultured in RPMI-1640 medium (Sigma-Aldrich, #R8758). This was supplemented with 10% v/v iron fortified foetal calf serum (Sigma-Aldrich, #C8056), 2% v/v penicillin-streptomycin (Sigma-Aldrich, #P4333) and 1% v/v amphotericin B solution (Sigma-Aldrich, #2942). This supplemented medium is hereafter referred to as complete culture medium (CCM). A series of T175 culture flasks (ThermoFisher Scientific, Roskilde, Denmark, #10246131) containing 50 mL of CCM were each seeded with 10^6^ cells and incubated in a 5% CO_2_ atmosphere in humidified incubator at 37°C. The CCM was changed on days 2, 4 and 7 post-seeding and cells were harvested when their density reached approximately 0.5x10^6^ cells mL^-1^. The cell suspensions were transferred to 50 mL centrifuge tubes (Sigma-Aldrich, #CLS430791) and centrifuged at 1200 rpm for 4 minutes in an Heraeus Megafuge 16 centrifuge equipped with a 3655-swinging bucket rotor (ThermoFisher Scientific). The supernatants were discarded, the cell pellets pooled and then resuspended in a small volume of CCM. A 100 μL sample of the final cell suspension was removed for viability estimation.

### Glass transitions

Differential scanning calorimetry (DSC) measurements were carried as described previously [6, 7] on cell pellets or cryoprotectant solutions using a power compensation calorimeter (Diamond, Perkin Elmer LLC, Norwalk, CT, USA) equipped with a liquid nitrogen cooling accessory (CryoFill, Perkin Elmer). Temperature calibration was performed using cyclohexane (crystal-crystal transition at -87.1°C), mercury and gallium (melting points at -38.6°C and +29.8°C, respectively).

For cellular measurements, approximately 10^8^ Jurkat cells in 10% DMSO (v/v) in CCM were pelleted at 13,000 g for 10 minutes, then transferred to a 2-mL Eppendorf tube and pelleted again twice at 16,000 g for 5 minutes, with the supernatant discarded between each cycle. Approximately 25 mg of the Jurkat cell pellet were placed in 50 μL Perkin Elmer DSC aluminium pans and sealed.

Additionally, some non-cellular solutions were examined. Cryoprotective solutions consisting of bovine albumin (Sigma-Aldrich, #A2153) suspended in CryoStor10 (Sigma-Aldrich, #C2874), a proprietary cryopreservation medium containing 10% DMSO, at incremental concentrations from 0 to 50% wt/wt were scanned. This models the intracellular macromolecular environment in a simplified binary solution, and can shed some light on the cryoprotective effects of serum [12].

For both cellular and non-cellular experiments, an empty pan was used as a reference. Different linear cooling rates of 1, 2, or 10°C min^-1^ were applied between 20°C and -150°C and samples were then warmed to 20°C at 10°C min^-1^.

Glass transition temperatures (Tg’,°C) and specific heat capacities (Delta Cp, J g^-1^°C) were calculated from heat flow data recorded during warming [6, 7]. Their derivatives versus temperature were calculated to better visualise glass transitions with a peak in output identified as different from the inherent noise of the machine when the first derivative of the heat flow reached a delta value of at least 2.5 mW s^-1^. Where the end of the glass transition event overlapped with ice melting and appeared as a shoulder in the derivative of the heat flow, the second derivative was calculated to identify the peak value. Ultimately, glass transition events were thus characterized by different values: i) the onset of the first derivative of heat flow that can be considered as the temperature of initial molecular mobility during heating; ii) the half extrapolated of heat flow and the maximum of the first derivative of heat flow, corresponding to the midpoint temperature of the glass transition; iii) the endset of the first derivative of heat flow that was considered as the temperature of initial loss of molecular mobility during cooling. Results were obtained from at least three separate cooling-warming cycles.

### Membrane lipid phase transition, ice nucleation and ice melting

In parallel to the DSC cellular measurements, a small portion of the cell pellet was used to measure membrane lipid phase transition by Fourier transform infrared spectroscopy (FTIR). For that, a Nicolet Magna 750 FTIR spectrometer (Thermo Fisher Scientific, Madison, WI, USA) equipped with a mercury/cadmium/telluride (MCT) detector and a variable temperature cell holder (Specac Ltd., Orpington, UK) were used. The cell sample was sandwiched between two calcium fluoride windows of 32.0 mm diameter by 3.0 mm thickness (ISP Optics, Riga, Latvia). These translucent windows held the sample in place, while allowing the passage of infra-red light required for measurements. The sample was then mounted in the Specac cell holder.

The procedure followed for analysis was as described by Gautier et al. [13]. Briefly, FTIR spectra were recorded in the mid-IR region from 4000 to 900 cm^-1^ at a 4 cm^-1^ resolution, from 32 co-added scans using Omnic software (version 7.1, Thermo Fisher Scientific). They were acquired at 45 second intervals during cooling from 37°C to -50°C and warming to 60°C, at rates of 2°C min^-1^ while continuously purging the optical bench with dry air (Balston, Haverhill, MA, USA). A type-K thermocouple was placed as close as possible beside the sample to provide continuous monitoring of temperature throughout the experiment.

ASpIR software (Infrared Spectra Acquisition and Processing, INRA, Thiverval-Grignon, France) was used to identify the position of infrared vibration bands of interest–the symmetric CH_2_ stretching vibration (νCH_2_) and the bend-libration H_2_O combination band. The position of the νCH_2_ vibration, located at approximately 2850 cm^-1^ and providing a measure for the conformational order of fatty acids chains of membrane phospholipids [14, 15], was identified as the peak maxima of the inverted second-order derivative of each raw FTIR spectrum. A sigmoid function was then fitted to the νCH_2_ temperature plots. Its first-order derivative was calculated, and the maximum of this derivative was taken as the lipid phase transition temperature upon cooling (membrane solidification, Ts) and warming (membrane melting, Tm). The bend-libration H_2_O combination band located between 2150 and 2220 cm^−1^ was used to determine ice nucleation and fusion in the extracellular compartment. During cooling, this band shifts extensively from 2150 to 2220 cm^−1^ due to liquid-to-solid phase transition of water, and the reverse occurs during warming [13]. The onset temperatures of these band shifts were therefore used as ice nucleation and ice melting temperatures, respectively. One run was carried out per independent sample, resulting in three measurements.

### Cryopreservation protocol

A suspension of Jurkat cells was prepared in CCM containing 10% v/v DMSO (Sigma-Aldrich, #D4540) at 3x10^6^ cells per mL and 1 mL aliquots added to 2 mL cryovials (Corning, sourced from ThermoFisher Scientific, #13429798). Vials were cooled at 1°C min^-1^ in a VIA Freeze controlled rate freezer (Asymptote, GEHC, Cambridge, UK). At defined endpoint temperatures between 4°C and -100°C, 5 vials were removed from the controlled rate freezer and plunged directly into liquid nitrogen (LN). Vials were stored below -140°C for at least 24 h before thawing.

For thawing, vials were removed from cold storage, placed into a dry SC2 VIA Thaw for vials (Asymptote, GEHC) and thawed for approximately 3 minutes. Once thawed (confirmed visually by lack of ice crystal), the vial contents were mixed to ensure homogeneity of the suspension and 25μL were placed into each well of two columns of a 96 well plate (ThermoFisher Scientific, #10212811), resulting in approximately 75,000 cells per well before cryopreservation damage is taken into account. 200 μL of CCM was added to each well to both allow the cells to culture and dilute the DMSO concentration below 1%. Five vials were included on each plate with edge columns filled with cell-free CCM to minimise edge effects.

The plates were cultured in a 5% CO_2_ atmosphere in a humidified incubator at 37°C, and assessed at 24, 48 and 72 h post-thaw culture.

### Viable cell counts and metabolic activity

Viable cell counts were made using fluorescein diacetate (FDA, Sigma-Aldrich, #F7378). 1 μL was added to each assessed well of the multi-well plate set up post-thaw and incubated in the dark at room temperature for 20 minutes. Total viable cell count was measured at 473 nm_ex_ /512 nm_em_ and calculated using a Cytell Imaging System (GEHC, Amersham, UK).

FDA is taken up by cells and hydrolysed into a fluorescent product by intracellular esterases, giving a measure only of cells which retain some metabolic capability.

Active respiratory metabolism of cells was estimated using Resazurin sodium salt (Sigma-Aldrich, #7017). Resazurin sodium salt evaluates the ability of cells to reduce the non-fluorescent resazurin to the fluorescent resorufin. This measurement indicates relative health of the cell; as background activity by non-viable cells can give low readings, a long incubation time of 3 h was chosen. Resazurin sodium salt was diluted in CCM to a final concentration of 0.005% w/v. 100 μL was added to each well under study with the multi-well plate incubated for 3 h at 37°C in a 5% CO_2_ humidified incubator. Plates were read in a fluorescence microplate reader (Tecan, Männedorf, Switzerland) with Magellan software (version 7.2, Tecan) at 560 nm_ex_ /612 nm_em_. Wells containing CCM and Resazurin sodium salt without cells were used to provide background values to be subtracted from the cell measurements.

### Statistical analysis

Data is presented as mean ± standard deviation (SD) throughout, unless otherwise stated in the text. Repeated measurements (indicated as n) were carried out on independent biological samples experiencing the same conditions unless otherwise stated. Means were compared in a pairwise fashion in R (version 3.4.2) [16] using the R Commander 2.4–1 package [17], and *P* values < 0.01 were considered significant.

## Results

### Membrane lipid phase transition, ice nucleation and ice melting

**Fig 1:**
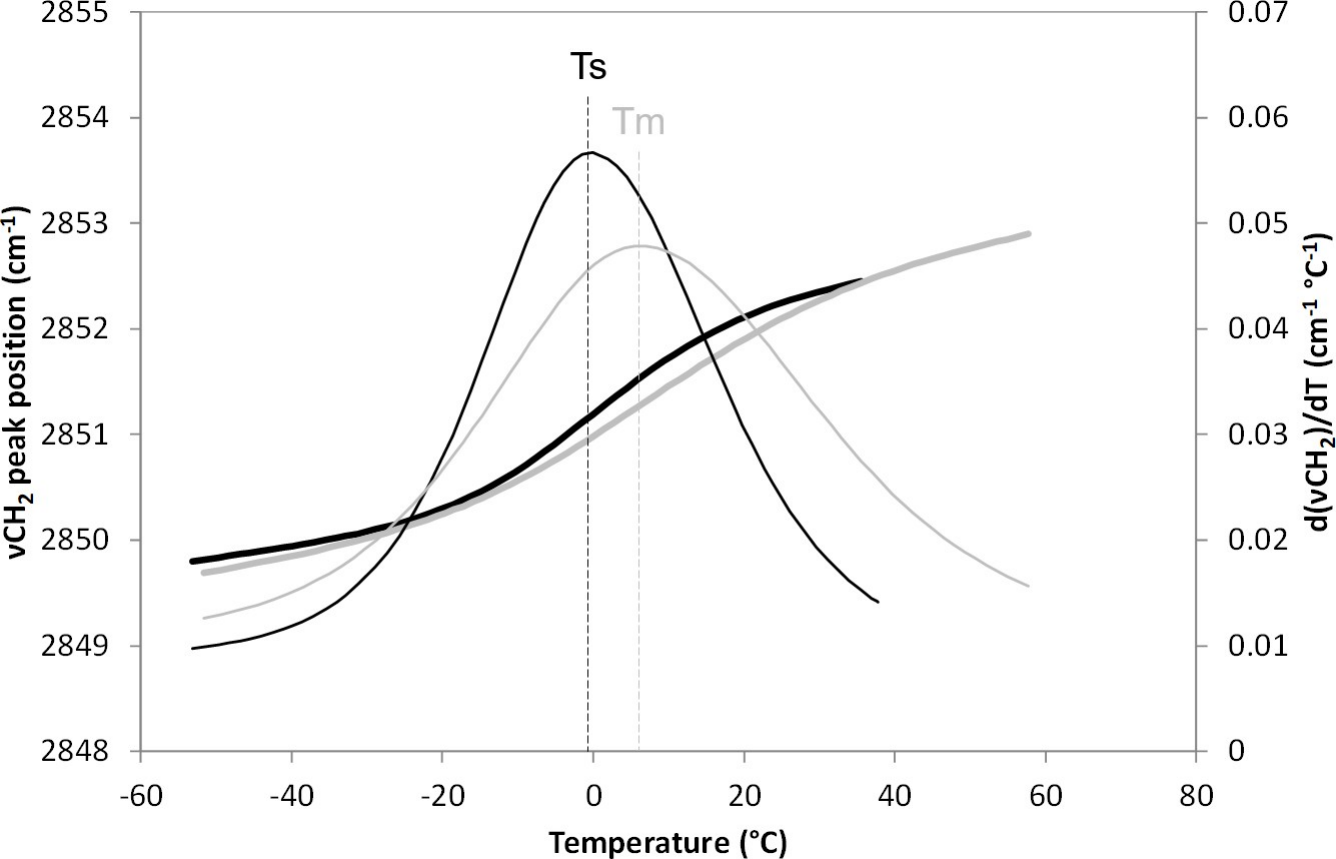
Membrane lipid phase transition of Jurkat cells identified by following the position of the symmetric CH_2_ stretching vibration (νCH_2_) of membrane fatty acids chains by Fourier transform infrared spectroscopy. Measurements were carried out during cooling from 37°C to -50°C (thick black) and warming to 60°C (thick grey) at rates of 2°C min^-1^. Derivatives of these curves were calculated (thin lines) and their maxima were taken as the membrane lipid phase transitions: solidification on cooling (thin black, Ts), observed at -1.0°C ± 0.8°C and melting on warming (thin grey, Tm), observed at 5.8°C ± 0.2°C (n = 3).

The first cellular, physical transition observed during cooling of a suspension of Jurkat cells in DMSO occurred within the cell membrane (Fig 1). The data indicate that membrane lipids, typically in a fluid and disordered state at temperatures compatible with cell growth, have moved to an ordered gel state upon cooling (black lines on Fig 1). The midpoint of this transition is defined as the membrane lipid solidification temperature (Ts) and appeared at -1.0°C ± 0.8°C. Extracellular ice nucleation occurred at -7.7°C ± 1.4°C. Both transitions were reversed on warming, ice melting being observed at -3.4°C ± 0.5°C, and the melting temperature of the lipid membrane at 5.8°C ± 0.2°C (grey lines on Fig 1). The raw data behind the means were included in S1 Table as supplementary information. The curve recorded during warming did not perfectly overlap that recorded during cooling, indicating some hysteresis in the membrane lipid thermotropic transition.

### Glass transitions

**Fig 2:**
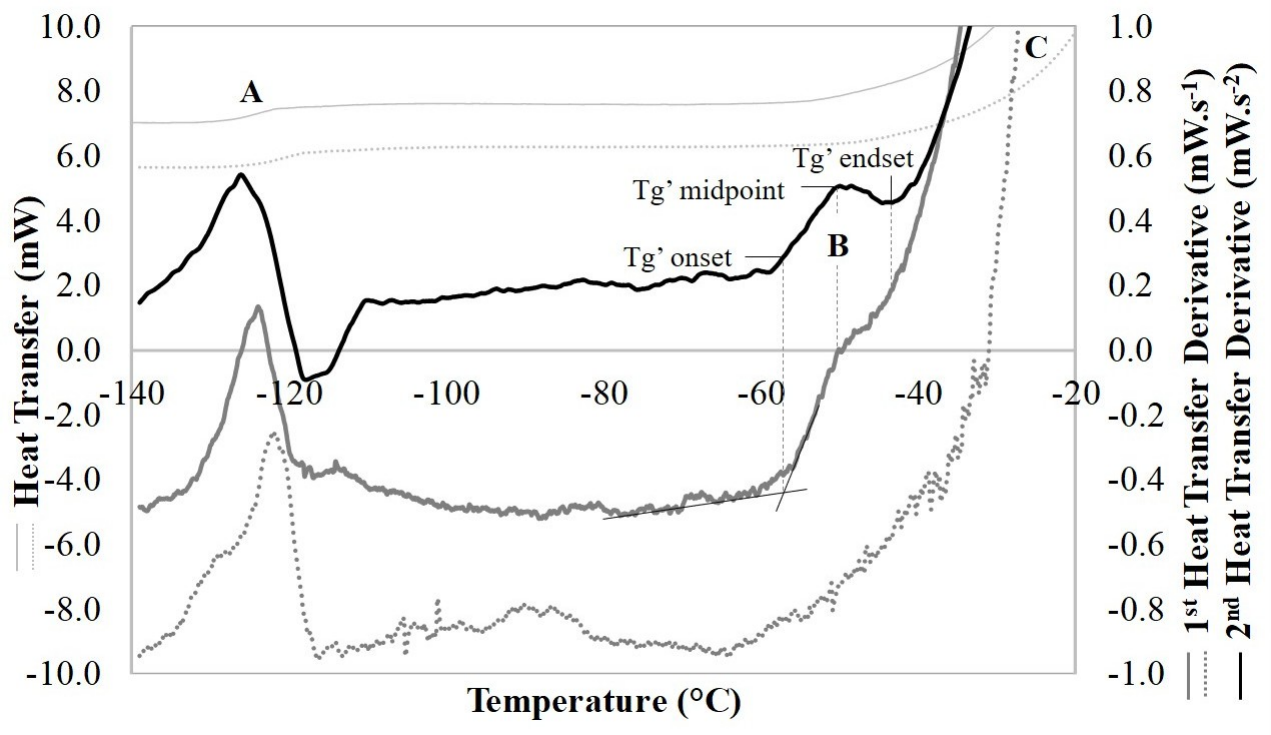
**A typical DSC trace of Jurkat cells (solid lines) and cell-free supernatant (dotted lines) during warming, and their first-order (grey) and second-order (black) derivatives.** Both samples were separated by centrifugation from a suspension of Jurkat cells in culture medium with 10% (v/v) dimethyl sulfoxide. They were initially cooled at 2°C min^-1^ to -150°C. Three distinct thermal events are apparent: the extracellular glass transition (A), the intracellular glass transition (B), and bulk melting of the extracellular solution (C). Note that transition B, characterized by its onset, maximum and endset, is absent in the DSC trace of the cell-free supernatant.

In samples initially cooled at 1°C min^-1^ to -150°C, ice nucleation in the extra-cellular compartment occurred between -6°C and -9°C. An endothermic event (event A, Fig 2) was observed by DSC during warming at -123°C, representing the midpoint of the glass transition of the extracellular medium (DMSO, Tg’e). An endothermic event was observed on further warming (event B, Fig 2), with an onset, midpoint and endset temperatures of -61.4°C ± 1.8, -53.0°C ± 0.7, and -46.9°C ± 1.3°C, respectively (average of 5 different biological samples ± SD; see S2 Table for the raw dataset). Bulk melting of the sample was accompanied by a large endothermic event (event C on Fig 2), observed to peak at -4°C. DSC measurements of cell-free supernatants (CCM supplemented with 10% v/v DMSO in the absence of cells) were also performed. No thermal events other than the extracellular vitrification (-123°C) and bulk melting of the sample were observed.

**Fig 3:**
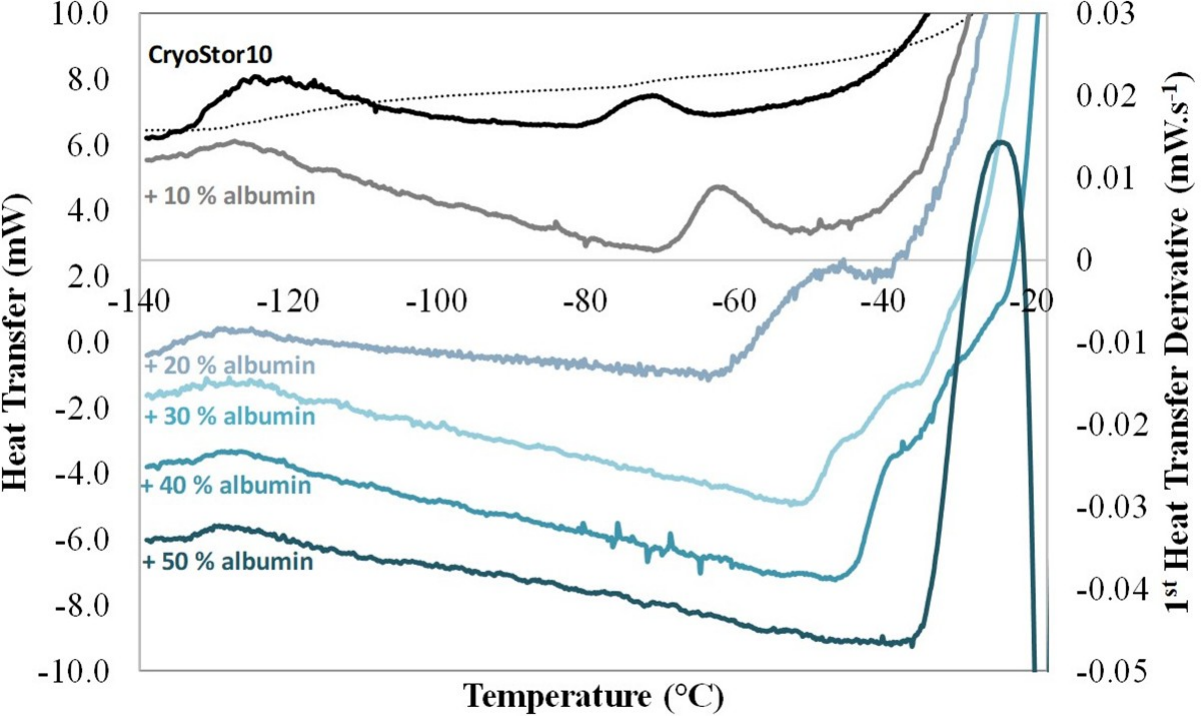
First-order derivatives of the DSC traces of mixtures of CryoStor10 and serum albumin during warming at 10°C min^-1^ following cooling at -10°C min^-1^. The following percentages, by weight, of albumin dissolved in CryoStor10 are identified by trace colour and are: black, 0%; grey, 10%; grey-blue, 20%; light blue, 30%; blue, 40%; dark blue, 50%. The raw DSC trace of CryoStor10 is also included (dotted black line).

DSC of slowly frozen samples of CryoStor10 exhibited an endothermic band centred at -122.5°C ± 0.6°C (n = 3), the typical glass transition of DMSO (Table 1, Fig 3 and Table for the raw dataset). Another endothermic event was observed with its endpoint located at -70.0°C ± 0.4°C. Both peaks may either reflect two consecutive thermal events of a single glass transition where the lower value represents the glass transition temperature of the freeze concentrated phase [18, 19] and the higher value represents the softening temperature at which the system exhibits an observable deformation (viscous flow in real time) under its own weight [20], or they may reflect the fact that this cryoprotective mixture consists of two phases that are not fully miscible. Adding increasing amounts of protein (albumin) to CryoStor10 (without cells) led the Tg’ of DMSO to disappear and a single endothermic event corresponding to Tg’ appeared at higher temperatures. The endset of this transition gradually and significantly increased with protein concentration (*P* < 0.01), reaching up to -21.9°C ± 0.6°C for a 50% wt. protein solution in CryoStor10 (Table 1, Fig 3 and S3 Table for the raw dataset).

**Table 1:** Glass transitions characterized by onset, midpoint and endset temperatures in mixtures of albumin suspended in CryoStor10 and measured by DSC upon warming (n = 3 ± SD).

	Tg’ onset (°C)	Tg’ midpoint (°C)	Tg’ endset (°C)
**CS10—peak #1**	-133.0 ± 1.4	-122.5 ± 0.6	-118.3 ± 5.2
**CS10—peak #2**	-80.4 ± 2.1 ^a^	-72.9 ± 1.5 ^a^	-70.0 ± 0.4 ^a^
**CS10 + 10% albumin**	-70.0 ± 3.6 ^b^	-62.3 ± 2.5 ^b^	-55.9 ±1.2 ^b^
**CS10 + 20% albumin**	-58.6 ± 0.8 ^c^	-45.9 ± 1.0 ^c^	-42.3 ± 0.9 ^c^
**CS10 + 30% albumin**	-50.0 ± 0.5 ^d^	-44.9 ± 0.3 ^c^	-38.3 ± 0.8 ^d^
**CS10 + 40% albumin**	-43.7 ± 0.6 ^e^	-38.3 ± 0.6 ^d^	-26.6 ± 0.6 ^e^
**CS10 + 50% albumin**	-34.2 ± 0.5 ^f^	-23.1 ± 1. 5 ^e^	-21.9 ± 0.6 ^f^

Different letters represent significantly different values following pairwise comparisons of means (*P* < 0.01).

### Biological transitions: Endpoint experiments

**Fig 4:**
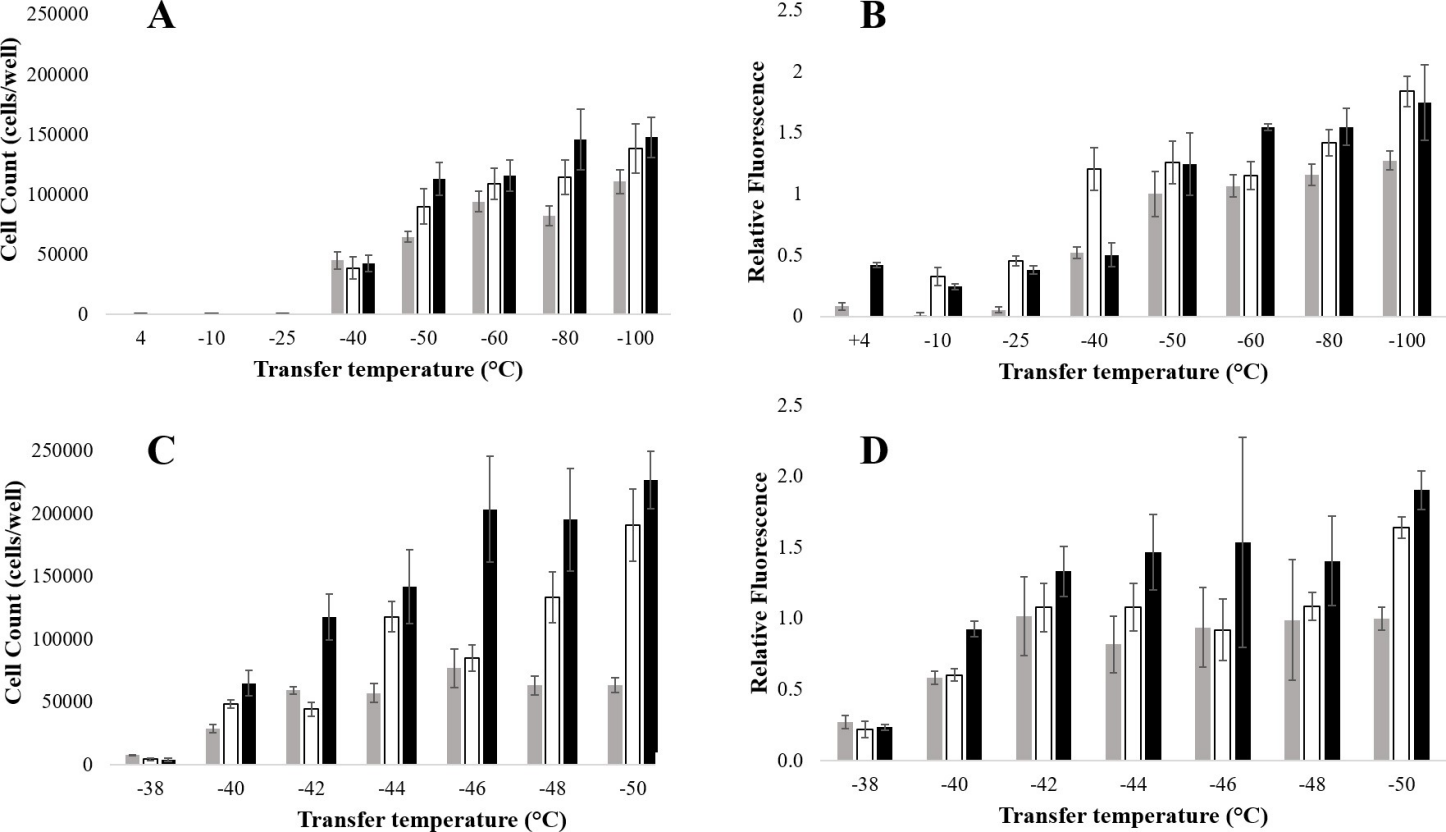
**Viable cell count (A, C), and metabolic activity (B, D) of Jurkat cells cooled down at 1°C min**^**-1**^ **to separate endpoints before plunging into liquid nitrogen.** Viable cell count was measured through fluorescein diacetate staining and metabolic activity was evaluated through the reduction of resazurin to the fluorescent resorufin, at 24 h (grey), 48 h (white), and 72 h (black), post thaw (n = 5 ± SD). For metabolic activity, fluorescent intensities were normalised to 1 at the -50°C, 24 h time point.

Cell suspensions were cooled at a slow controlled rate down to a range of temperatures before being transferred to liquid nitrogen. In the first series of experiments, endpoint temperatures between 4 and -100°C prior to plunging into LN were examined. At -25°C or higher temperatures, high cell mortality post-thaw and no proliferation up to 72 h post-thaw was observed (Fig 4A and S4 Table for the raw dataset). Conversely, when samples were transferred to LN from -50°C or below, a high number of cells survived (mean 8.8x10^4^ ± 1.9x10^4^ viable cells per well 24 h post-thaw) and were able to proliferate (mean 13.1x10^4^ ± 2.3x10^4^ per well 72 h post-thaw). All samples plunged into LN at ≤ -50°C had significantly higher (*P* < 0.01) metabolic activity and higher viable cell number than those transferred at temperatures ≥ -25°C at all timepoints measured. The initial density of cells was 7.5x10^4^ cells per well immediately post-thaw. When transferred to LN from -40°C, an intermediate number of cells survived (4.5x10^4^ ± 0.7x10^4^ 24 h post-thaw), but they were not able to proliferate (4.2x10^4^ ± 0.7x10^4^ 48 h post-thaw). The metabolic activity data post-thaw followed a similar pattern (Fig 4B and S5 Table for the raw dataset).

In experiments with a 2°C interval in transfer temperature between -38°C and -50°C, low viable cell numbers and metabolic activity were obtained for cells that were transferred into LN from -38°C, with no proliferation up to 72 h of culture, (Fig 4C and 4D, and S6 and S7 Tables for the corresponding raw datasets). A trend was observed with lower temperature of transfer resulting in higher cell viability, samples transferred at -50°C had significantly higher (*P* < 0.01) viable cell numbers than when transferred at -44°C, 48 and 72 h post-thaw (Fig 4C and S6 Table). A similar pattern was observed for metabolic activity values (Fig 4D and S7 Table).

## Discussion

**Fig 5:**
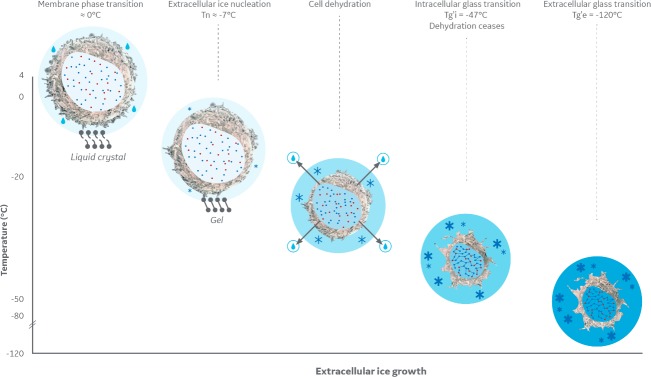
Schematic of the physical transitions occurring during cryopreservation of a Jurkat cell in the presence of dimethyl sulfoxide. As temperature decreases (from left to right), the cell membrane first undergoes a liquid-crystal to gel phase transition, with its midpoint at approximately 0°C. At approximately -7°C ice starts to nucleate in the extracellular compartment and with ice crystal growth the extracellular solute concentration increases. Intracellular water flows out of the cells in response to the osmotic gradient. As cell dehydration takes place, the intracellular compartment becomes more and more crowded (the blue and red dots symbolising various macromolecules), until it undergoes a glass transition, starting at -47°C. Intracellular macromolecules now form a network (linked dots) analogous to a colloidal glass, that behaves as a molecular sieve in which nanopores still remain liquid. Upon further cooling, the extracellular compartment and the intracellular nanopores undergo a glass transition at -123°C (darkening of the blue shade of the intra- and extracellular compartments). Below this temperature, molecular mobility ceases. Jurkat cell images adapted from Walter et al. [24].

The membrane lipid phase transition observed approximately 5°C above the extracellular compartment’s ice nucleation and melting temperature (Fig 1) is known not to affect cell viability substantially in other mammalian cell types [21]. It is reversible, although with a slight hysteresis, highlighting an increased lipid conformational order during warming than during cooling [22, 23]. During the further, imposed slow cooling in the presence of extracellular ice, the Jurkat cells were subjected to dehydration as extracellular freeze concentration withdrew water from the cell. The endothermic event subsequently occurring (event B, Fig 2) was ascribed to an intracellular glass transition (Tg’i, event B, Fig 2). It should be noted that this vitrification signal detected in the cell suspensions by DSC, was absent from cell-free supernatants and so represents a cellular event. Finally, the extracellular medium vitrified at -123°C. Fig 5 schematically illustrates these sequential events taking place in a suspension of Jurkat cells during equilibrium cryopreservation in the presence of DMSO.

It is postulated here that the observed endothermic event in the cell samples is the intracellular glass transition of a maximally freeze concentrated intracellular compartment [6, 7]. Several alternative interpretations of this phenomena can be ruled out:

The observed event is not related to lipids as i) the phase transition of membrane lipids occurred at temperatures close to zero as determined by FTIR spectroscopy, and ii) Jurkat cells contain no bulk storage lipids. This is also not melting of intracellular ice since the high cell viability obtained on thawing is incompatible with intracellular ice formation during cooling, according to Mazur’s two-factor hypothesis [24]. Further, direct observations of human lymphocytes during cooling showed that intracellular ice does not occur at rates of cooling less than 5°C min^-1^ [25]. Finally, in the present work, the cooling rate applied is too slow and the thawing rate too fast to enable devitrification events upon warming [26].

Being able to identify the occurrence of this colloidal-like glass transition in slowly cooled cells in DMSO by a complementary method to DSC would nevertheless be ideal. However, such a potential analytical or observation method is not conceivable today because of the low temperatures involved and the presence of ice. The data presented here are, to our knowledge, the first direct evidence of an intracellular glass transition in a mammalian cell suspension undergoing conventional cryopreservation, and which corresponds to biological outcomes.

As ice nucleation is a stochastic phenomenon that may hinder or modify the occurrence of other thermal events, DSC thermograms are commonly analysed upon warming [6, 7]. The endset of a thermal transition identified on warming can thus inversely be thought of as an onset during cooling. Therefore, during cooling, Jurkat cells would start to undergo an intracellular glass transition at a temperature around -47°C (Tg’i endset). This value agrees with the key biological event identified to occur between -40°C and -50°C, with viable cell counts and metabolic activity of the cells being completely removed when transfer to LN occurs above -40°C and being relatively intact below -50°C (Fig 4).

The midpoint of this transition was located at -53.0°C ± 0.7°C, close to the -50°C value reported by Fonseca et al. as the intracellular glass transition of bacteria in the presence of DMSO [7]. Studying model solutions of cryoprotectant with protein, mimicking different intracellular protein contents, allowed to foresee the behaviour of Tg’i as a function of intracellular protein content (Fig 3). Higher Tg’i for higher intracellular protein concentrations agrees with the higher protein content in bacteria compared to mammalian cells [27].

As described by Zhou et al. [8] for osmotically compressed cells, the glass transition of the intracellular compartment resulting from an increased packing density of intracellular macromolecules (proteins, polysaccharides, nucleic acids) and organelles is analogous to a colloidal glass transition. Cryoconcentration being equivalent to the osmotic compression of cells in terms of water removal and increased intracellular packing density, the endothermic event occurring in slowly cooled Jurkat cells at -47°C could thus also be described as similar to a colloidal glass transition. Macromolecules in suspension in the cytoplasm of cells are believed to form this colloidal-like glass [8, 28], which can be thought of as a mesh of immobilised macromolecules due to the large increase of viscosity associated with glass transition. This glass would lock up some water, DMSO and other small molecules (ionic species, metabolites) in between the mesh of glassy macromolecules, that would remain liquid when the colloidal-like glass forms. The formation of an intracellular glass would also make the membrane stiffer as it is composed of proteins with cytoplasmic and membrane domains. While small molecules can move within this intracellular colloidal-like glass, interaction with the extracellular environment is severely curtailed. Cells can no longer dehydrate and so are in their maximally dehydrated state offering maximum protection from intracellular ice formation.

Once the cell interior has vitrified the cell would thus be expected to be osmotically unresponsive. A colloidal glass behaves like a molecular sieve, in the case of *E*. *coli* [9] allowing the free passage of small molecules such as glucose (MWt 180) whilst restricting the diffusion of bigger ones such as green fluorescent protein GFP, a 24kDa protein with a cylindrical shape of length of 4.2 nm and diameter of 2.4 nm [29]. As proteins typically range in size from 2–10 nm [30], average pore sizes in the dehydrated state are in the order of only a few nm. Formation of an intracellular glass during freeze concentration would not be expected to significantly modify the osmolality of the residual intracellular solution or its volume. The concentration of the freeze concentrated DMSO would be expected to be 50% with a viscosity in the order of 100 mPa.s [30]. This residual intracellular solution would be expected to change state (either to vitrify or nucleate as ice) during subsequent cooling. The formation of an intracellular glass does not remove the problem of intracellular ice formation, but it changes its geometry from a single intracellular compartment to multiple nanopores within a colloidal-like glass. Upon this transition from the microporous to the nanoporous (< 10 nm pore diameter) volume regime, ice nucleation is therefore severely depressed within pores. The melting point can be further suppressed to temperatures well below that of homogeneous freezing temperature of bulk water, as has been shown with nanoporous silica [31], zeolites [32], silica gels and also airborne nanodroplets [33]. In the case of an intracellular colloidal-like glass the degree of this effect may also depend on the influence of highly concentrated solutes which may be expected to encourage vitrification of the small volumes within the pores. The importance of the intracellular glass transition to the survival of cells at low temperatures is that this transition does not involve the change in volume and density that accompanies ice nucleation and freezing, and inhibits bulk intracellular ice formation. A cell in which the intracellular compartment freezes is subject to mechanical stresses, which are frequently lethal, and a primary cause of cell death during cryopreservation [1]. However, a cell in which an intracellular glass forms behaves effectively as a solid and retains its internal structure and integrity. The fact that these cells have a high viability on thawing demonstrates that the intracellular glass transition is not a lethally damaging event.

The temperature range between intracellular and extracellular glass transition will be determined by the protein content of the cell, as well as its dehydration on cooling. Higher protein concentration, and greater dehydration will result in a higher Tg’i (Fig 3). If a freezing medium contains a high level of protein (such as media with high fractions of albumin or serum), this medium too may undergo a glass transition before the DMSO event at -123°C.

In this paper we have focused on the physical transitions which occur during conventional cryopreservation of Jurkat cells in 10% DMSO (schematically summarised in Fig 5) and have significant implications for its success. Typically, cells are cooled at a controlled, slow rate to -80°C or below, before being transferred into liquid nitrogen or its vapour. This work provides a scientific basis to show that this transfer should not commencebefore approximately -50°C (to take account of thermal fluctuations during transfer), at least for Jurkat cells in 10% DMSO, and lower temperatures such as -60°C will provide a robust thermal buffer in the event of warming during transfer. While only Jurkat cells were considered in this study the results are likely to apply to other cultured cell lines cryopreserved in the presence of DMSO, due to similar relevant physical characteristics. It should also be noted that the formation of an intracellular glass during cooling would not, by itself, ensure the long-term stability of a cell. Water and solutes such as ionic species and metabolites would be mobile within pores in the intracellular glass and degradation reactions could still occur there, or between the extracellular compartment and the cell membrane. Consequently, long-term stability in the frozen state is achieved below both the extracellular Tg’ and the Tg’ of the aqueous compartment in the intracellular glass, i.e. below -123°C.

## Supporting information

S1 Table Raw data behind the phase transition temperatures of Jurkat cell samples identified by FTIR spectroscopy. Membrane lipid solidification (Ts) and ice nucleation (Tn) temperatures upon cooling, and temperatures of melting (Tm) of ice and membrane lipid upon warming.
(XLSX)

S2 Table Raw data behind the intracellular glass transition (Tg'i) of Jurkat cell samples measured by DSC, characterised by onset, maximum and endset tempeartures. (XLSX)

S3 Table Raw data behind the glass transitions of mixtures of albumin suspended in CryoStor10 (CS10) measured by DSC, characterised by onset, maximum and endset temperatures. (XLSX)

S4 Table Raw data behind the viable cell count of Jurkat cells cooled down at 1°C min-1 to separate endpoints before plunging into liquid nitrogen. Viable cell count was measured through fluorescein diacetate staining.
(XLSX)

S5 Table Raw data behind the metabolic activity of Jurkat cells cooled down at 1°C min-1 to separate endpoints before plunging into liquid nitrogen. Metabolic activity was evaluated through the reduction of resazurin to the fluorescent resorufin. Fluorescent intensities were normalised to 1 at the -50°C, 24 h time point.
(XLSX)

S6 Table Raw data behind the viable cell count of Jurkat cells cooled down at 1°C min-1 to zoomed 2°C interval endpoints before plunging into liquid nitrogen. Viable cell count was measured through fluorescein diacetate staining.
(XLSX)

S7 Table Raw data behind the metabolic activity of Jurkat cells cooled down at 1°C min-1 to zoomed 2°C interval separate endpoints before plunging into liquid nitrogen. Metabolic activity was evaluated through the reduction of resazurin to the fluorescent resorufin. Fluorescent intensities were normalised to 1 at the -50°C, 24 h time point.
(XLSX)
